# Prophylactic antimicrobials may not be needed to prevent surgical site infection after skin biopsy: a retrospective study

**DOI:** 10.1186/s13756-022-01077-z

**Published:** 2022-02-16

**Authors:** Yuko Akiyama, Yuta Norimatsu, Yuki Ohno

**Affiliations:** 1 Department of Dermatology, JR Tokyo General Hospital, 2-1-3 Yoyogi Shibuya-ku, 151-8528 Tokyo, Japan; 2 Department of Dermatology, University of Tokyo Graduate School of Medicine, Tokyo, Japan

**Keywords:** Antibiotics, Biopsy, Prophylactic antimicrobials, Surgical site infection

## Abstract

**Background:**

Two types of skin biopsies are routinely performed in dermatology: excisional and punch biopsies. A punch biopsy is a relatively low-risk procedure for surgical site infections (SSIs) because of the shallow wound depth and short operative time. In Japan, prophylactic antimicrobial agents are often used after skin biopsies due to lack of consensus, and there is no mention of antimicrobial use after skin biopsies in Japanese guidelines. In this study, we investigated whether prophylactic antibiotic use after punch biopsies reduces the risk of SSI development.

**Methods:**

Cases of punch biopsy performed in our dermatology department during a one-year period from April 2018 to March 2019 were included retrospectively. The cases were divided into a group with and another without prophylactic antimicrobial use after biopsy.

**Results:**

A total of 75 cases of punch skin biopsy were reviewed. There were no cases of wound infection after punch biopsy in any of the groups. The number of years of experience of the physicians in the group that used antimicrobials was significantly higher than that in the group that did not use antimicrobials (*P* < 0.0001).

**Conclusions:**

Our result suggests that the incidence of SSI in punch biopsies without prophylaxis seems to be low. However, further research is needed due to the small number of cases in this study.

## Background

Surgical site infections (SSIs) occur within 30 days after surgery and are categorized according to their depth into three types: superficial, deep, and organ/body cavity SSIs. Some of the risks for SSI are patient-related factors, such as age, nutritional status, diabetes, smoking, obesity, remote site infection, microbial flora, altered immune status, and duration of hospital stay before surgery. Other risk factors for SSIs are site-related factors, such as hand washing during surgery, skin disinfection, preoperative shaving, operation time, antibiotic administration, operating room ventilation, inappropriate disinfection of instruments, foreign bodies at the surgical site, drains, and tissue damage [1]. Therefore, to prevent SSIs, surgeons should pay attention to hand washing, skin disinfection, operating room ventilation, temperature control, and aseptic manipulation [2].

Skin biopsy is a frequently performed procedure in dermatology [3]. There are two types of skin biopsies: excisional and punch. An excisional biopsy is used to remove large masses or lesions, while a punch biopsy is widely used for the excision and sampling of small nevi [4]. A punch biopsy is considered a relatively low-risk procedure for SSI because of its short surgical time and shallow wound depth, which limits any infection to the superficial skin layers [1, 3].

Regarding infection prevention protocols, the American College of Surgeons and the Infectious Diseases Society of America do not recommend the use of antimicrobial agents after skin biopsies [2]. However, the Japanese Society for Chemotherapy and the Japanese Association for Surgical Infectious Diseases do not list recommendations on the dermatological use of antimicrobial agents in the practical guidelines for the prevention of postoperative infection. In Japan, dermatologists use antibacterial agents for the prevention of SSIs. However, no guidelines are established on the use of prophylactic antibiotics after routine punch biopsies.

In the field of dermatology in Japan, no study has been reported on the difference in SSI rates with and without prophylactic antibiotic use after punch biopsies. In this study, we investigated whether prophylactic antibiotic use after punch biopsies reduces the risk of SSIs.

## Methods

Seventy-five cases of skin punch biopsies performed in our department over a one-year period from April 2018 to March 2019 were included in this retrospective study.

The cases were divided into two groups: those in whom prophylactic antibiotics were used and those whom they were not used.

Individuals who underwent excisional biopsies were excluded from this study because an excisional biopsy is not standardized in our hospital. The presence or absence of signs of infection, such as redness, heat, and pus drainage, was assessed during subsequent outpatient visits.

All patients received a skin biopsy in the operating room of the Department of Dermatology, JR Tokyo General Hospital.

We defined the absence of SSI occurrence as the absence of SSI on examination after 7 days and the absence of patient return visits over the next 30 days.

The evaluation items were suture, punch biopsy major axis, site of biopsy, years of experience as a doctor, age, sex, body mass index, brinkman index, liver dysfunction, diabetes mellitus, liver dysfunction, kidney dysfunction, diabetes mellitus, hypertension, kidney dysfunction, hyperlipidemia, hypertension, cancer, hyperlipidemia, immunosuppressant use, albumin.

Statistical analysis was performed using Prism 8 (GraphPad Software Inc., La Jolla, CA, USA), and the data were analyzed with the Mann-Whitney U and Chi-square test (Fisher’s exact tests). Statistical significance was set at *P* < 0.05.

## Results

The results are presented in Tables 1, 2, 3 and 4.

**Table 1:** Surgery related characteristics of the patients

Surgery related characteristics of the patients	Antibacterial drug used (n = 27) (mean ± SD^a^)	No antibacterial drug used (n = 48) (mean ± SD)	*P* value
Suture (+:−)	23:4	19:29	0.0002^†^
Punch biopsy major axis (3 mm:4 mm:5 mm)	5:9:13	13:20:15	0.3396^†^
Site of biopsy (face:trunk:inguinal:extremity)	8:4:2:13	16:12:2:18	0.6306^†^
Years of experience as a doctor	8.519 ± 6.417	5.042 ± 3.287	< 0.0001^‡^

^a^Standard deviation

+: positive

−: negative

Fisher’s exact test or Mann–Whitney U test was used for statistical analysis

^†^Chi-square test (Fisher’s exact test)

^‡^ Mann–Whitney U test

**Table 2:** Characteristics of the patients

Patient characteristics	Antibacterial drug used (n = 27) (mean ± SD^a^)	No antibacterial drug used (n = 48) (mean ± SD)	*P* value
Age	55.11 ± 20.61	59.85 ± 17.83	0.3869^‡^
Sex (male:female)	14;13	30;18	0.4651^†^
Body mass index (kg/m^2^)	21.04 ± 2.689	23.24 ± 5.003	0.0501^‡^
Brinkman Index	396.9 ± 294.2	682.2 ± 454.8	0.0961^‡^

^a^Standard deviation

Fisher’s exact test or Mann–Whitney U test was used for statistical analysis

^†^Chi-square test (Fisher’s exact test)

^‡^ Mann–Whitney U test

**Table 3:** Medical history of the patients

Medical history	Antibacterial drug used (n = 27) (mean ± SD^a^)	No antibacterial drug used (n = 48) (mean ± SD)	*P* value
Liver dysfunction (+:−)	0:19	2:37	> 0.9999^†^
Diabetes mellitus (+:−)	1:18	9:23	0.0695^†^
Kidney dysfunction (+:−)	2:16	2:36	0.5866^†^
Hypertension (+:−)	4:16	12:24	0.3646^†^
Hyperlipidemia (+:−)	4:16	10:24	0.5326^†^
Cancer (+:−)	10:14	8:25	0.2484^†^
Immunosuppressant use (+:-)	0:22	3:29	0.2621^†^

Fisher’s exact test or Mann–Whitney U test was used for statistical analysis

+: positive

−: negative

^a^Standard deviation

^†^Chi-square test (Fisher’s exact test)

**Table 4:** Blood test

Blood test (normal range)	Antibacterial drug used (n = 27) (mean ± SD^a^)	No antibacterial drug used (n = 48) (mean ± SD)	*P* value
Albumin (g/dL) (3.9–5.1)	4.153 ± 0.4658	4.033 ± 0.5377	0.2803^‡^

^a^Standard deviation

Fisher’s exact test or Mann–Whitney U test was used for statistical analysis

^‡^ Mann–Whitney U test

Risk factors were evenly distributed in both groups. The number of years of experience of the physicians in the group that used antimicrobials was significantly higher than that in the group that did not use antimicrobials (*P* < 0.0001). More sutures were performed in the antimicrobial group than in the non-antimicrobial group (*P* = 0.0002). Details of the antimicrobial agents included amoxicillin in 3 cases, cefaclor in 21 cases, and cefdinir in 3 cases. Postoperative antibacterial drug usage days are 4.593 ± 2.258.

There was no difference in the development of SSI between the two groups.

No patient had SSI in both groups.

## Discussion

This study showed that the incidence of SSI in punch biopsies without prophylaxis seems to be low.

Immunodeficiency is not said to be a risk for SSI. In the present report, about 1/4 of the patients had cancer or were on immunosuppressive drugs, but they did not have SSI, these results are consistent with a previous report [5].

Prophylactic antibiotics are recommended for patients with high-risk cardiac disease, diabetes mellitus, and for a defined group of prosthesis patients at high risk for hematogenous total joint infection. It is also recommended if the surgical site is infected or if the surgery involves destruction of the oral mucosa. To prevent SSIs, antibiotics may be indicated for lower extremity and inguinal surgery, wedge resection of the lips and ears, nasal skin flaps, skin grafts, and for patients with extensive inflammatory skin diseases [6, 7]. In this report, there were also a certain patients who underwent skin biopsies to the extremities and inguinal area, and patients with diabetes, but none of them developed SSI. It was suggested that prophylactic antimicrobials are not necessary for dermatology punch biopsies as recommended by the American College of Surgeons and the Infectious Diseases Society of America [2]. Common risk factors for skin infections include damage to the skin barrier function, skin inflammation due to eczema or radiation therapy, impetigo, prior infections such as ringworm, and lymphedema [8]. Furthermore, lymphedema, chronic venous insufficiency, peripheral circulatory disturbance, and deep vein thrombosis have been identified as the common risk factors for recurrent cellulitis [9]. However, unlike in other countries, hypertension, hyperlipidemia, hypoalbuminemia, and lymphedema may be risk factors for skin infections in Japanese patients [10]. It has been reported that Japanese women with cellulitis are more likely to be carriers of carcinoma compared to Japanese men, who have a more typical risk background for cellulitis [11]. A low body-mass index was also reported in the Japanese cases. The difference in the risk factors for cellulitis between Japan and other countries may be due to the difference in the risk factors associated with women [10, 11].

Our results suggest that prophylactic antimicrobial therapy after skin biopsy is not necessary even in Japanese patients with different medical backgrounds. In this study, no patient developed adverse events; however inappropriate use of antimicrobial agents may cause *Clostridium difficile* infection, anaphylactic shock, and medical economic losses [12, 13]. A decrease of approximately 10% in the total number of antimicrobial agents prescribed to outpatients could reduce the number of *C. difficile* community-acquired infections by 17% [14]. In addition, from the perspective of reducing medical costs, if prophylactic antimicrobial agents were not administered, our department alone could reduce the medical cost by about 150 dollars annually. Approximately 3000 general hospitals in Japan that offer dermatology services use convalescent care beds, with an estimated annual cost of approximately about 435 thousand dollars for these services. Furthermore, since punch biopsies are routinely performed in outpatient dermatology clinics (in approximately 12,000 facilities in Japan), the medical costs that can be reduced by refraining from prophylactic antimicrobial administration is considerably high.

Additionally, dermatologists have been reported to be more inclined to give prophylactic antimicrobials [15]. Our results also show that the more years of experience dermatologists have, the more likely they are to prescribe prophylactic antimicrobials. Evidence-based medicine began to attract attention in Japan in 1997. Until then, empiric therapy with mainly third-generation cephems was recommended for skin infections. For example, some textbooks used by Japanese dermatologists still list cefdinir and faropenem as the first-line treatment. However, with the introduction of the Japanese Society of Infectious Diseases certified specialist system in 1998 [16], and the super-rotation system in 2004 (Medical Practitioner’s Act, 2013; unpublished data), education on infectious diseases in Japan has progressed, and the appropriate use of antimicrobial agents has become widespread in recent years. In 2011, the World Health Organization (WHO) adopted the theme, ‘measures to prevent the development of drug-resistant strains of bacteria, for the World Health Day. Furthermore, in 2015, the WHO announced the Global Action Plan, which advocated the need for global treatment recommendations. In response, Japan created the National Action Plan on Antimicrobial Resistance to combat drug resistance as a national project in 2016 [17]. As a result, infection control teams consisting of physicians, nurses, pharmacists, and clinical laboratory technicians became engaged not only in infection prevention activities but also in activities to promote the appropriate use of antimicrobial agents. As one of the measures, the use of certain antimicrobial agents (broad-spectrum antimicrobial agents, anti-MRSA drugs, etc.) now require notification and an issuance of a permit, which have contributed to curbing the inappropriate use of antimicrobial agents. Currently, this restriction is limited to intravenous antimicrobial agents used in the hospital setting. However, it is also necessary to appropriately restrict the use of oral antimicrobial agents in the outpatient setting. Based on the above discussion, we believe that the use of prophylactic antimicrobial agents during skin punch biopsies should be carefully considered to decrease the adverse effects caused by antimicrobial agents, development of drug-resistant bacteria, and medical cost.

## Conclusions

We believe that the findings of this study make a significant contribution to the dermatology field because there are currently no specific guidelines for the use of antibiotics during skin punch biopsies in Japan. Furthermore, these findings may encourage Japanese dermatologists to reduce the use of prophylactic antibiotics and in punch biopsies, which may potentially reduce medical cost and adverse events caused by antimicrobial agents. However, due to the small number of cases in this study, further research is needed.

## Data Availability

The datasets generated and/or analyzed during the current study are available from the corresponding author on reasonable request.

## Abbreviations

SSI: Surgical site infection
WHO: World Health Organization
